# MicroRNA profiling and identification of let-7a as a target to prevent chemotherapy-induced primordial follicles apoptosis in mouse ovaries

**DOI:** 10.1038/s41598-019-45642-w

**Published:** 2019-07-03

**Authors:** C. Alexandri, B. Stamatopoulos, F. Rothé, Y. Bareche, M. Devos, I. Demeestere

**Affiliations:** 1 Research Laboratory in Human Reproduction, Faculty of Medicine, Université Libre de Bruxelles (ULB), Brussels, Belgium; 2 Laboratory of Clinical Cell Therapy, Jules Bordet Institute, Université Libre de Bruxelles (ULB), Brussels, Belgium; 3 Breast Cancer Translational Research Laboratory, Institute Jules Bordet, Université Libre de Bruxelles (ULB), Brussels, Belgium

**Keywords:** Molecular medicine, Cancer

## Abstract

Cancer treatments as cyclophosphamide and its active metabolites are highly gonadotoxic leading to follicle apoptosis and depletion. Considering the risk of subsequent infertility, fertility preservation is recommended. Beside the germ cells and gametes cryopreservation options, ovarian pharmacological protection during treatment appears to be very attractive. Meanwhile, the advances in the field of oncology have brought microRNAs into spotlight as a potential feature of cancer treatment. Herein, we investigated miRNAs expressions in response to chemotherapy using postnatal-day-3 (PND3) mouse ovaries. Our results revealed that several miRNAs are differently expressed during chemotherapy exposure. Amongst them, let-7a was the most profoundly downregulated and targets genes involved in crucial cellular processes including apoptosis. Thus we developed a liposome-based system to deliver the let-7a mimic in whole PND3 ovaries *in vitro*. We showed that let-7a mimic prevented the upregulation of genes involved in cell death and reduced the chemotherapy-induced ovarian apoptosis, suggesting that it can be an interesting target to preserve ovarian function. However, its impact on subsequent follicular development has to be further elucidated *in vivo* using an appropriate delivery system. In this study, we demonstrated that miRNA replacement approaches can be a useful tool to reduce chemotherapy-induced ovarian damage in the future.

## Introduction

The mammalian ovary is a highly dynamic organ characterized by a well-controlled process able to regulate the activation and development of follicles which ensure both reproductive and endocrine ovarian functions^1,2^. The pool of primordial follicles, also known as “ovarian reserve” is defined at birth and its number declines with increasing age until the onset of menopause^3,4^.

Cancer and oncological treatments can disrupt the normal process of folliculogenesis and damage the ovarian reserve, leading to a high risk of future infertility^5^. Anticancer treatments including chemotherapy as Cyclophosphamide (Cx) can cause primordial follicle activation, apoptosis and vascularization damage leading to premature ovarian insufficiency^6^. Hence, several Oncological Societies strongly recommend informing young patients about the risk of infertility after treatment, giving them the opportunity to preserve their fertility. The most effective and established methods to preserve fertility are oocyte and embryo cryopreservation while alternative experimental options entail ovarian tissue cryopreservation and *in vitro* maturation of immature oocytes^7–9^. However, these methods are invasive and cannot be applied to all of the patients. Therefore, there is a need of developing new strategies to preserve fertility in female cancer survivors. The pharmacological protection of the ovaries aiming to reduce the gonadotoxic effects of chemotherapy appears to be a very attractive and promising alternative.

Meanwhile, the breakthroughs in the field of oncology revealed that microRNAs (or miRNAs or miR) are interesting targets with therapeutic potential. MiRNAs are short non-protein-coding RNA molecules of 21–23 nucleotides acting as major post-transcriptional gene regulators by hybridizing with a target mRNA sequence (with total or partial complementation) resulting in translational repression or degradation^10^. According to recent studies, miRNAs regulate a wide range of cellular processes such as growth, differentiation and apoptosis and they play a crucial role in tumorigenesis and cancer evolution^11^. As therapeutic tools, miRNAs can be used to modulate and increase the sensitivity of the neoplastic cells to chemotherapy, while it has been proved that they are themselves modulated by chemotherapy^12^. Accordingly, we hypothesized that miRNAs might also be useful to reduce chemotherapy-off target toxicity in healthy cells.

The advances in the field of genomics and the application of new-age technologies revealed that these small non-coding molecules are essential for the mammalian ovarian function and they have a key role in follicular growth, atresia, hormonal production and cross-talk between oocyte and cumulus cells^13–16^. Recently, Xiao and their colleagues revealed that amniotic fluid stem cells containing miR-146a and miR-10a reduced chemotherapy-induced ovarian damage in mice^17^. Similarly, exposure to mesenchymal stem cells transfected with miR-21 reduced granulosa cells (GCs) apoptosis induced by chemotherapy^18^.

In this study we used postnatal-day-3 (PND3) mouse ovaries which mainly consist of primordial follicles as a model for chemotherapy-induced ovarian damage. We aimed to evaluate the effect of chemotherapy on miRNAs expression in ovarian follicles at early stages of development as they reflect the ovarian reserve in women. We identified and validated miRNAs which can be used to develop novel ovarian protective agents against chemotherapy damage. Amongst them, let-7a mimic was tested as potential ovarian protective approaches against chemotherapy-induced apoptosis.

## Results

### MiRNAs are differently expressed in mouse postnatal ovaries in response to 4-hydroperoxycyclophosphamide (4-HC)

**Figure 1:**
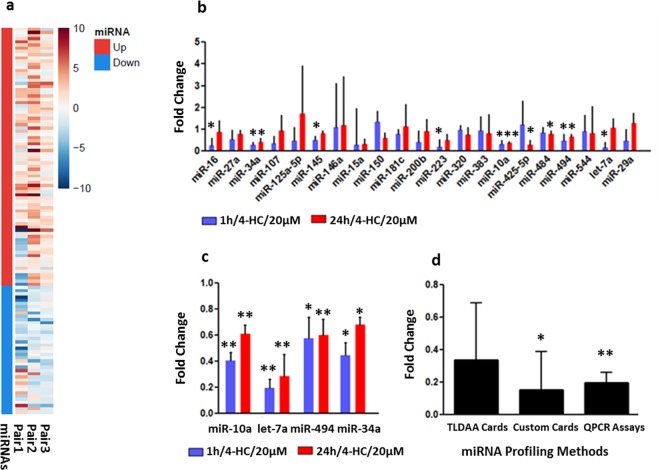
(**a**) Heatmap representation of 121 miRNAs expression in PND3 ovaries exposed to 4-HC for 1 h compared to control using Rodent TLDA Cards: 81 were upregulated (FC > 1.5) while the 40 were downregulated (FC < 0.5) after 1 hour exposure to 4-HC/20 µM. The experiments were repeated 3 times and the data analysis was performed by comparing miRNAs expression in paired groups (control-chemo exposed). The miRNAs and their expression levels are listed in the Supplementary Table S1. (**b**) Validation of the expression levels of selected miRNAs. Fold change of selected miRNAs which are differently expressed in PND3 ovaries after 1 h (N = 10) and 24 h (N = 6) exposure to 4-HC/20 µM compared to unexposed ovaries using TaqMan Custom Cards. The selected miRNAs and their expression levels are listed in the Supplementary Table S2. (**c**) Fold change of miR-10a, let-7a, miR-494 and miR-34a after individual QPCR validation in PND3 ovaries after 1 h and 24 h exposure to 4-HC/20 µM compared to unexposed ovaries. A minimum of 9 experiments have been performed per tested miRNAs. (**d**) Fold change of let-7a throughout all the miRNA expression profiling methods: TLDA cards (N = 3 pairs), Custom Cards (N = 10 pairs), QPCR Assays (N = 10 pairs). Fold change represents the geometric mean. The error bars represent the standard error. Significant differences compared to control are expressed as *p < 0.05, **p < 0.001 using paired t-test.

The miRNAs expression profile was assessed by Rodent TLDA cards (Applied Biosystems™) in 3 individual experiments. For each experiment, the control and chemo-exposed PND3 ovaries (1 h/4-HC/20 µM) were compared by pairs. From the 384 genes present on the card, 245 genes were expressed according to the data analysis parameters (Cut-off Ct > 32). Among them, 74 were stable, 81 (33%) were upregulated (FC > 1.5), 40 (16%) were downregulated (FC < 0.5) and 51 presented an undetermined profile (Fig. 1a, Supplementary Table S1). The comparison between control and 4-HC exposed samples in each experimental pair revealed that miRNAs are themselves modulated in PND3 ovaries during chemotherapy exposure.

### miR-16, miR-34a, miR-145, miR-10a, miR-223, miR-494 and let-7a are significantly down-regulated after 1 hour exposure to 4-HC

In order to validate the differently expressed miRNAs, a Custom TLDA card with 21 selected miRNAs and 3 control genes was designed. The 21 miRNAs of the Custom TLDA card were selected according to their expression profile (up- or down-regulation) on TLDA cards and their biological function. According to the literature, most of the selected miRNAs are involved in pathways of cell proliferation, DNA damage response and apoptosis^19–21^. Moreover, it has been shown that some of these miRNAs play an important role in ovarian function and development^22^. We assessed the expression levels of the selected miRNAs in a total of 16 PND3 ovarian pairs (control-chemotherapy exposed). The results confirmed that miR-16, miR-34a, miR-145, miR-10a, miR-223, miR-494, let-7a and miR-34a, miR-10a, miR-425-5p, miR-484, miR-494 are differentially expressed in PND3 ovaries after 1 and 24 hours exposure to 4-HC (20 µM), respectively (p < 0.05) (Fig. 1b and Supplementary Table S2). By monitoring chemotherapy impact at two time points (1 and 24 h exposure) we showed that the change in miRNAs expression was not only a short-term effect but rather was maintained after 24 hours as their expression levels did not return to basal levels. These results confirmed that chemotherapy has an impact on miRNA transcriptional regulation.

Individual TaqMan Assays further validated the miRNA expression levels of 4 miRNAs which were found to be significantly expressed on Custom Cards (miR-10a, let-7a, miR-494, miR-34a) (Fig. 1c). The QPCR results revealed that let-7a was the most downregulated miRNA and its expression profile was consistent throughout all validation methods (Fig. 1d).

### let-7a targets essential genes involved in DNA damage and apoptosis

**Figure 2:**
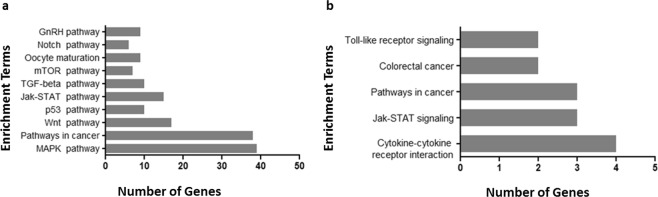
Pathway enrichment analysis on DAVID database on predicted by TargetScan (**a**) and validated targets by miRTarBase of let-7a (**b**). Let-7a targets molecules which are involved in pathways of DNA damage response, apoptosis and cell proliferation.

According to the MGI database (informatics.jax.org), let-7a targets more than 1000 predicted genes. However, in order to be able to manage this huge network of predicted genes interactions, we focused on the experimentally validated miRNA targets reported on miRTarBase while TargetScan was used to identify miRNA predicted interactions. The Pathway Enrichment Analysis performed on DAVID database revealed that let-7a can regulate the expression of genes which are involved in important cellular pathways like JAK/STAT pathway, cancer, DNA damage, apoptosis and cell cycle (Fig. 2).

### mimic miRNA can be efficiently transfected into whole PND3 ovaries

**Figure 3:**
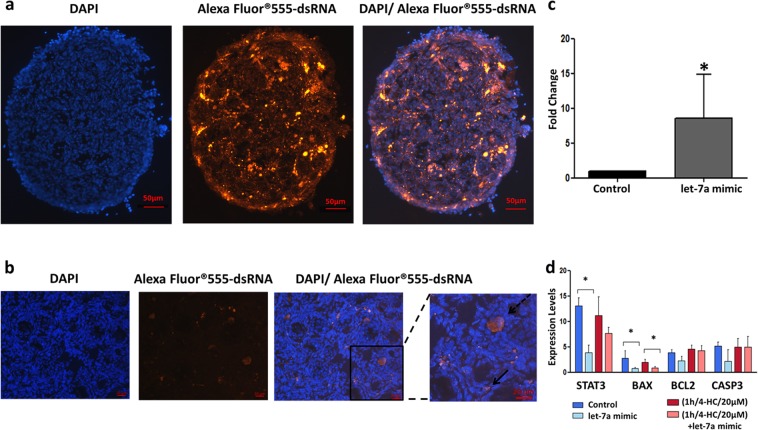
Evaluation of transfection’s efficiency in PND3 ovaries. (**a**) Representative images of PND3 ovary, after 2 days of transfection with Alexa Fluor® 555-labeled, dsRNA/ Lipofectamine RNAiMax. PND3 ovarian sections (10 µm) showed nuclear labelling with Hoechst (blue) and Alexa Fluor® 555-labeled, dsRNA (red). The merged images indicate that the dsRNA was been successfully transferred into the ovaries. (**b**) Magnified images indicate that after transfection the labelled-dsRNA is located mainly in stroma and granulosa cells (bold arrow) and in the oocyte of some follicles (dashed arrow). (**c**) Expression levels of let-7a after transfection with let-7a mimic using liposome-delivery system. The fold change of let-7a is significantly higher in PND3 ovaries compared to control conditions (N = 3), *p-value < 0.05. The error bars represent the standard error. (d) Expression levels of genes involved in apoptosis in four different groups: control, chemotherapy alone (1 h/4-HC/20 µM), let-7a mimic alone (let-7a mimic), chemotherapy + let-7a mimic ((1 h/4-HC/20 µM) + let-7a mimic). STAT3 and BAX are significantly downregulated in the control + let-7a group (N = 5). BAX is significantly downregulated in the chemo + let7-a group (N = 8).

Given that let-7a is downregulated in PND3 ovaries after exposure to chemotherapy, let-7a mimic was delivered into the whole PND3 ovaries to prevent chemotherapy damage. The transfection of the whole organ was performed to avoid any damage and follicular activation due to ovarian manipulation or follicles isolation. In order to evaluate the transfection’s efficiency we used a fluorescent labeled dsRNA and the Lipofectamine RNAiMax as a carrier. After transfection, we detected a fluorescent signal throughout the ovarian sections. The liposome-mediated delivery was efficient even though the fluorescent labelled-dsRNA was not uniformly distributed due to the ovarian morphology, shape and heterogeneous cell population (Fig. 3a,b, Supplementary Fig. S1).

To confirm the efficacy of Lipofectamine-let-7a mimic transfection, the expression levels of its corresponding miRNA were assessed. While the basal expression level was variable, let-7a expression was significantly higher after transfection in all the experiments (Fig. 3c).

**Figure 4:**
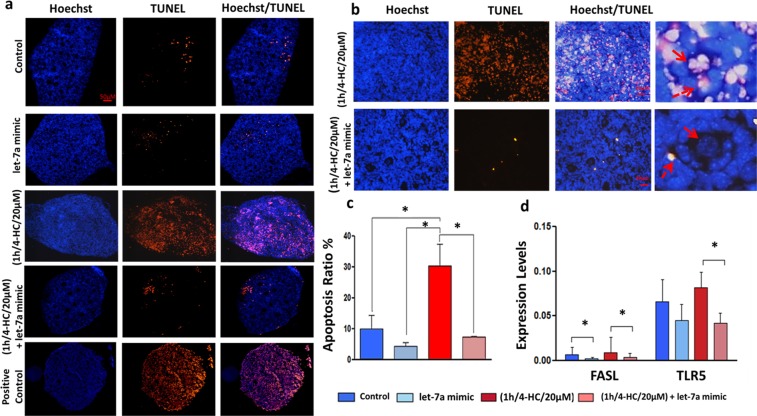
Apoptosis evaluation in PND3 ovaries with or without treatments. (**a**) Ovarian sections (5 µm) show nuclear labelling with Hoechst (blue) and apoptotic cells /TUNEL positive (red). After 2 days in culture, apoptosis was observed in both non-chemo exposed groups; control and let-7a mimic but at the second group the apoptosis was lower. The apoptosis levels in ovaries treated with chemo (1 h/4-HC/20 µM) are comparable to positive control (DNAse induced damage). However, in chemo + let-7a mimic group apoptosis is much more lower. (**b**) Magnified images indicate that after chemotherapy exposure apoptosis is mainly induced in stroma and granulosa cells (dashed arrow) while oocytes are also affected (bold arrow). The transfection with let-7a mimic reduces this effect. (**c**) Quantification of apoptotic cells. The results are presented as the mean of three separated measurements (N = 3), the error bars represent the standard error, p < 0.05. (**d**) Expression levels of genes involved in apoptotic signalling pathways in 4 different groups: control, let-7a mimic, chemotherapy (1 h/4-HC/20 µM), chemotherapy (1 h/4-HC/20 µM) + let-7a mimic. The expression level of FASL is significantly reduced after transfection with let-7a mimic in both control and chemotherapy exposed ovaries (N = 7).The error bars represent the standard error, *p < 0.05.

**Figure 5:**
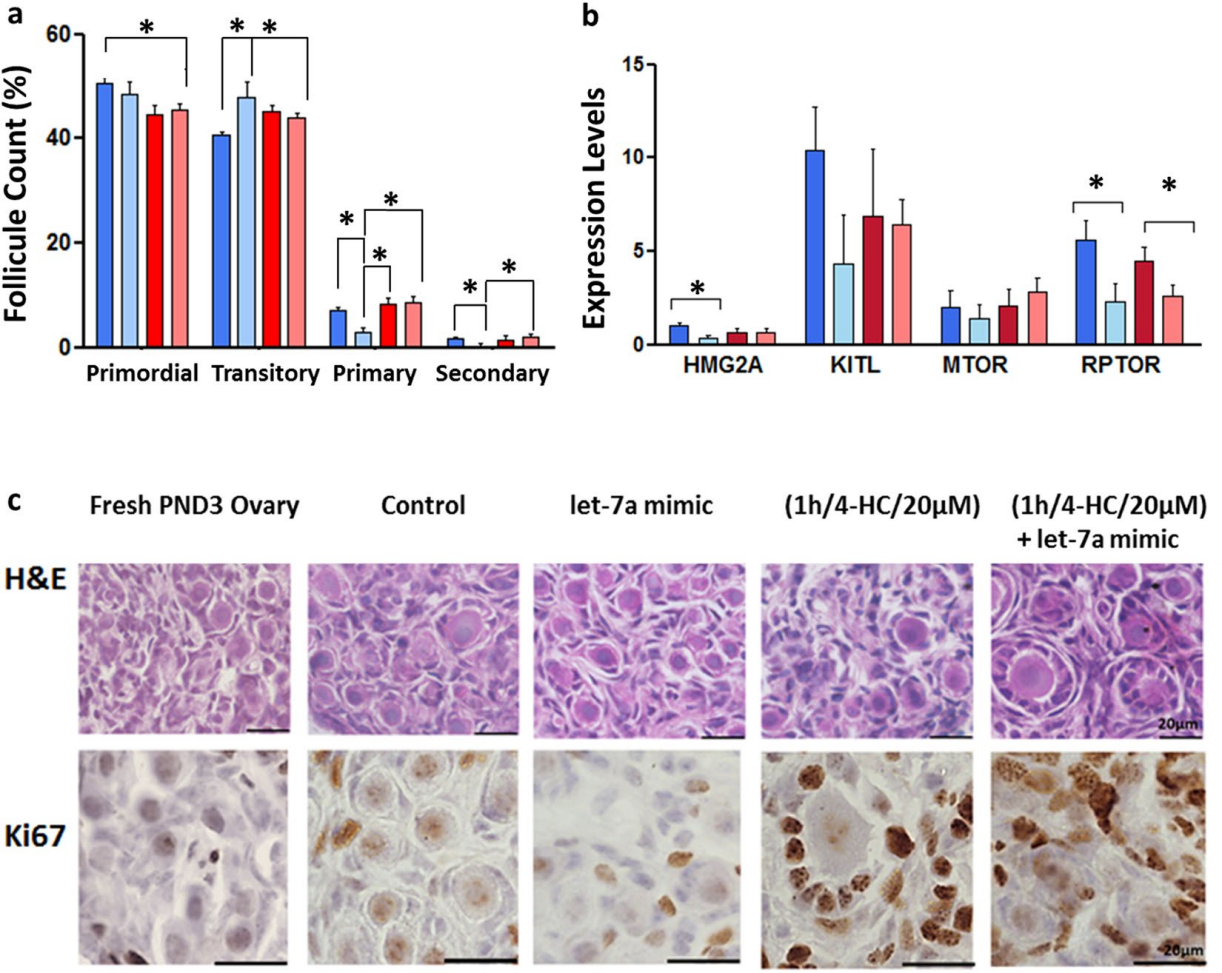
(**a**) The graph presents the percentage of follicles in different developmental stages in the 4 groups. The follicle counting was performed in three different experimental samples for each group (N = 3). The error bars represent the standard error, p < 0.05. (**b**) Expression levels of genes related to cell proliferation in different conditions. HMGA2 and RPTOR are significantly downregulated in the let-7a mimic group (N = 5). RPTOR is significantly down-regulated in (1 h/4-HC/20 µM) + let-7a mimic group (N = 6). The error bars represent the standard error, p < 0.05. (**c**) Haematoxylin & Eosin (H&E) staining and immunostaining of Ki67 on 5 µm sections of PND3 ovaries in different conditions. The active proliferative cells of the follicles (granulosa cells) are Ki67 positively stained.

To confirm the efficiency of the method, the expression of selected mRNA targets based on let-7 family predicted and validated targets like STAT3, FASL, TLR5 and HMGA2 was evaluated^23–25^. The expression of these genes was evaluated in 4 conditions: control, chemotherapy alone (1 h/4-HC/20 µM), let-7a mimic alone, chemotherapy + let-7a mimic (1 h/4-HC/20 µM/ + let-7a mimic). STAT3 showed a decrease after let-7a mimic transfection when compared to control (Fig. 3d). Moreover, FASL was downregulated in both let-7a transfected groups (associated with chemotherapy or not) while TLR5 showed a decrease after let-7a mimic transfection when it was exposed to chemotherapy (Fig. 4d). Last, HMGA2 was downregulated after let-7a mimic transfection but the difference reached significance only when let-7a mimic transfection was compared to control (Fig. 5b).

### Let-7a mimic transfection reduced chemotherapy-induced damage in PND3 mouse ovaries

To explore the protective effect of let-7a in PND3 ovaries exposed to chemotherapy, apoptosis was evaluated in the 4 conditions: control, chemotherapy alone (1 h/4-HC/20 µM), let-7a mimic alone, chemotherapy + let-7a mimic (1 h/4-HC/20 µM + let-7a mimic). After 2 days, the TUNEL assay confirmed that the apoptosis levels were decreased after transfection with let-7a mimic in both let-7a mimic alone and 1 h/4-HC/20 µM + let-7a mimic groups compared to control and chemotherapy alone groups respectively (Fig. 4a,b). Moreover, the expression of BAX and FASL decreased in the presence of let-7a mimic alone or in association with chemotherapy compared to control and chemotherapy alone groups, respectively (Figs 3d and 4d).

### Let-7a mimic transfection did not reduce follicular activation after chemotherapy exposure

The expressions of genes which are specifically involved in follicular activation (KITL and mTOR) were not significantly modulated by let-7a while others were significantly downregulated (RPTOR and HMGA2, p < 0.05) (Fig. 5b). After 2 days in culture, the follicle counting confirmed the spontaneous activation induced by culture conditions irrespective of the transfection with let-7a mimic. However, these results did not excluded a potential impact of let-7a mimic group may on follicular growth as the numbers of primary and secondary follicles are reduced in the transfected ovaries compared to the other groups (Fig. 5a,c).

## Discussion

Different miRNA-based therapeutics have been recently involved in clinical trials, especially in the field of oncology^26^. On the other hand, studies also indicate that miRNAs have a key role in follicle development as they can regulate the follicular growth, atresia and steroidogenesis. Hence, by studying the role of miRNAs in follicular response to toxic agents, we aim to develop new therapeutic tools for keeping follicles healthy during chemotherapy.

Given that there is no data available on the miRNAs expression in response to chemotherapy exposure in post-natal mouse ovaries, we first screened the miRNAs change during short exposure to 4-HC at a dose that induces toxicity without leading to massive cell death^27^. The present study is the first to report that miRNAs rapidly response to the changes of their microenvironment by altering their expression after chemotherapy-induced ovarian damage *in vitro*. Hence, by considering that miRNAs control multiple signalling pathways, even subtle alterations in their expression can have a major impact on biological processes such as follicle apoptosis and development. Consequently, double validation for the selected miRNAs revealed that let-7a was the most dysregulated miRNA. Let-7a presented a stable and profound downregulated profile in all miRNA screening assays, indicating that let-7a may has a key role in chemotherapy induced ovarian damage. Considering its biological importance; let-7a, lethal-7 (let-7) miRNA was one of the two first miRNAs discovered in *C*. *elegans*, as a major regulator of development^28^. However, its function in chemotherapy induced ovarian damage has not been studied yet. Moreover, the highly conserved nature of let-7 family makes let-7a an ideal candidate, as the results may lead to wider conclusions about other species. Regarding the implication of let-7 in cancer pathways, members of let-7 family have been characterized as tumour suppressors^29^. As the expression of let-7 is decreased in several types of cancer^30,31^ it is considered as a potential target for tumour therapy^29^. Moreover, the let-7 family appears to have a key-role in proliferation, survival and apoptosis of granulosa cells^32,33^.

In order to elucidate the role of let-7a in ovarian follicles in response to chemotherapy exposure, we performed gain-of function experiments using the let-7a mimic. After transfection of let-7a mimic, the expression levels of let-7a were significantly increased while targeted genes like HMGA2 (Highly Motility Group A2), STAT3 (Signal Transducer and Activator of Transcription 3) were significantly downregulated. These genes are important regulators of cell cycle, apoptosis and cell adhesion and their dysregulation can lead to tumorigenesis^24,25^. The TLR5 (Toll-like Receptor 5) which was also affected after mimic transfection, is involved in inflammatory responses and is expressed on the surface epithelium of healthy and cancer ovaries^34^. However, the mechanism of action of the miRNAs has to be taken into consideration in the interpretation of the results. It is known that miRNAs can bind to their target by perfect or imperfect complementarity leading to mRNA cleavage or translational inhibition^35^. Moreover, miRNAs regulate genes through different mechanisms leading to gene silencing that may not always be obvious at mRNA level^36,37^. Nevertheless, our results confirmed that the transfection with let-7a mimic was effective as we detected a downregulation at mRNA level of genes normally targeted by the endogenous let-7a.

In addition, the impact of let-7a upregulation on the expression profiles of apoptotic and cell-cycle related genes were evaluated in the presence of chemotherapy. We showed that FASL (Fas ligand) and BAX (BCL2-associated X protein) expression was significantly downregulated after let-7a mimic transfection, even in the presence of chemotherapy. FASL is a predicted target of let-7 family and it has been implicated in granulosa cell death during atresia. It is also involved in p53-mediated apoptosis^38^. Granulosa cells damage can lead to follicular death through bidirectional communication between oocytes and their surrounding cells^39^. Moreover, cyclophosphamide can induce mitochondrial-mediated apoptotic events and lead to upregulation of BAX expression^40^. In our study, BAX is downregulated after let-7a mimic transfection in the presence or absence of chemotherapy while it is not known as a direct target of let-7a. These results suggested that other molecules which are implicated in the regulation of BAX-mediated apoptosis are affected by let-7a upregulation. Therefore, the downregulation of BAX and FASL after transfection with let-7a seems to be essential in preventing cell-death.

Finally, the strong evidence confirming that let-7a can protect early stage follicles from apoptosis after chemotherapy-induced ovarian injury arises from TUNEL assay. The number of cells undergoing DNA-fragmentation or apoptosis in the group exposed to chemotherapy after let-7a mimic transfection was significantly reduced compared to the chemotherapy alone group. After 1 h of chemotherapy exposure, we identified apoptotic cells in both stroma and ovarian follicles (granulosa cells and oocytes). However, the transfection with the let-7a mimic can prevent this deleterious effect of chemotherapy. Furthermore, our results are consistent with previous study where the role of let-7 family in apoptosis has been reported. According to this study, the expression of let-7a/b/c/i was found to be downregulated while the let-7g was increased in atretic porcine follicles. Then, the functional analysis revealed that overexpression of let-7a/b/c/I by using miRNA-mimics, reduced the number of apoptotic granulosa cells compared to let-7g mimic which induced cell death^33^.

Genes related to follicle activation and cells proliferation are not specifically direct targets of let-7a like mTOR (Mammalian target of rapamycin), RPTOR (Regulatory-associated protein of MTOR complex 1) and KITL (Ligand for the receptor-type protein-tyrosine kinase KIT). Nevertheless, the overexpression of let-7a resulted in lower expression levels of the RPTOR, irrespective of chemotherapy exposure, while mTOR and KITL expressions were unchanged. RPTOR participates in PI3K/Akt/mTOR signalling pathway and regulates the function of mTORC1 which controls crucial cellular processes like growth, protein synthesis and activation of primordial follicles^41^. The increase in mTORC1 activity enhanced the activation of the p70 S6 kinase 1-ribosomal protein S6 (S6K1-rpS6) signalling that promotes follicle activation^42,43^. By reducing RPTOR expression level, let-7a can indirectly modulate the function of mTORC1 complex resulting in a control of primordial follicle activation. However, the difference of mTOR expressions were not significant between groups but the gene is mainly regulated at post-transcriptional level by phosphorylation^44^. Using histological analysis, we detected growing follicles in both control and treated groups as the follicular growth is accelerated during *in vitro* culture compared to the *in vivo* conditions. The follicle counting confirmed that the transfection with let-7a mimic did not slow down the growth and the development of the follicles. Despite results are encouraging, the real impact on the ovarian reserve, follicle growth and oocyte maturation competence need to be further evaluated in long term using *in vivo* model.

According to the ASCO’s 13th Annual Report, the breakthroughs in the field of oncology have converted cancer into chronical or even curable disease and cancer survivors have raised expectations for better quality of life including the possibility to procreate in the future. While pharmacological option to prevent gonadal damage during oncological treatment appears to be very attractive, the only molecule tested to protect ovaries from chemotherapy-induced damage is gonadotrophin-releasing hormone agonist, but its effectiveness remains controversial^45^. Our first monitoring of the miRNAs expression during chemotherapy exposure came up with promising results. We identified let-7a mimic as a molecule with anti-apoptotic properties which can be evolved into a potential ovarian protective molecule. Given that several RNA therapeutics are now on clinical trials, the increasing interest and knowledge on this therapeutic approach in oncology creates new perspectives in using miRNAs also as a fertility preservation option. However, there are several barriers before reaching clinical application. The most challenging part of miRNA-based therapeutics is to develop an effective delivery system which can target specifically the organs of interest without causing serious side effects or additional toxicity by triggering the immune system. We acknowledge that bridging the gap between the bench and the bench-side requires more improvements concerning the miRNA-delivery system, the targeted transfer into the ovaries, the toxicity and the immune-response of the recipient. To conclude, despite the fact that there is space for further development, these findings represents an encouraging starting point to bring miRNAs a step closer to clinical application for female fertility protection during oncological treatments.

## Methods

### Ethics statement

All the experimental procedures were approved by the local Animal Ethics Committee of “Université Libre de Bruxelles” and were performed in accordance with relevant guidelines and regulations.

### Animal

C57BL/6xCBAF1 hybrid mice at 3-days old (PND3) were used in all the experiments. Animals were maintained under standard controlled light and temperature-conditions and provided with food and water *ad libitum*.

### *In vitro* Culture of Mouse Ovaries

Female PND3 mice were sacrificed by decapitation. The ovaries, which consist mainly of primordial follicles, were harvested and collected in Leibovitz L-15 medium (Life Technologies, Belgium) supplemented with fetal bovine serum (FBS 10%), 1 mg/ml streptomycin and 6 mg/ml penicillin G (Sigma, Belgium). Whole ovaries were carefully cleaned from the surrounding tissues and placed in 24-well culture plates. Each ovary was cultured on a Millicell-CM filter membrane (Merck Millipore, Belgium), in 240 μl of DMEM F12 medium (Life Technologies, Belgium) supplemented with albumin and bovine serum (BSA) (1 mg/ml) (Sigma, Belgium), Albumax (1 mg/ml) (Life Technologies, Belgium), ascorbic acid (50 μg/ml), human transferrin (27.5 μg/ml), penicillin G (5IU/ml) and streptomycin sulphate (3.7IU/ml) (Sigma, Belgium).

To evaluate the miRNAs profile in response to chemotherapy exposure, a pool of PND3 ovaries was randomly split in two equal groups; one group represented the normal condition (control) and the other the chemotherapy-induced ovarian damage. Specifically, the ovaries were cultured at 37 °C in a humidified incubator with 5% CO_2_, in the presence or absence of 4-hydroperoxycyclophosphamide (4-HC, 20 µM) (Sigma, Belgium), during 1 or 24 hours. After culture, the ovaries were processed for miRNA extraction.

For mimic-RNA transfection experiments, four conditions were tested; culture alone (control), chemotherapy exposure (1 h/4-HC/20 µM), culture with let-7a mimic (let-7a mimic) and chemotherapy exposure + let-7a mimic (1 h/4-HC/20 µM + let-7a mimic).

### TaqMan Low Density Arrays (TLDA)

The miRNA expression profiles in PND3 ovaries (3 controls, 3 chemotherapy-exposed) were performed according to the manufacturer’s instructions using Rodent TaqMan® Low Density Arrays (TLDA type A card, Applied Biosystems, Belgium). For each experiment, PND3 ovaries were pooled from 3–4 mice from the same litter and randomly assigned in control and treated groups. The format of TLDA card type A focuses on 384 highly characterized miRNAs and includes candidate endogenous controls (U6, snoRNA135, snoRNA202, U87, Y1) and negative control assays (ath-miR). Briefly, RNA from PND3 ovaries was extracted using ReliaPrep™ miRNA Cell and Tissue Miniprep System (Promega, Netherlands) according to the manufacturer’s instructions including homogenization and DNAse treatment steps. The quantity and purity of RNA was determined using NanoDrop spectrophotometer (Thermo Scientific, Belgium) while the RNA Integrity Number (RIN) was measured by Agilent 2100 Bioanalyzer system. The samples were proceed directly to cDNA synthesis using the TaqMan® MicroRNA Reverse Transcription kit (Applied Biosystems, Belgium) and multiplexed Megaplex™ RT Primers (Applied Biosystems™) according to the manufacturer’s instructions. A step of pre-amplification was performed using 2.5 µl from the cDNA product, TaqMan® PreAmp kit (Applied Biosystems, Belgium) and Megaplex™ PreAmp Primers (Applied Biosystems, Belgium) according to the manufacturer’s instructions, to increase the sensitivity. The pre-amplified products were diluted in final volume of 100 µl Tris-EDTA buffer (pH8). The diluted product was mixed with TaqMan Universal PCR Master Mix® (Life Technologies, Belgium) and each sample was loaded into the 8 card’s ports (100 μl/port). The PCR reactions were performed using the ViiA 7 Real-Time PCR System with TaqMan Array Block (Life Technologies, Belgium).

### Custom TaqMan Array 384-well Cards

After TLDA-card screening, the expression levels of selected miRNAs were measured by TaqMan® Custom 384-well Cards (Applied Biosystems, Belgium). The Custom cards feature 24 targets in duplicates: snoRNA202, U87, U6, miR-10a, miR-15a, miR-16, miR-27a, miR-34a, miR-107, miR-125a-5p, miR-145, miR-146a, miR-150, miR-181c, miR-200b, miR-223, miR-320, miR-383, miR-425, miR-484, miR-494, miR-544, let-7a and miR-29a. Customs cards were processed similarly as TLDA cards.

### Individual TaqMan® Gene Expression Assays

Additional expression level validation was performed by real-time PCR with individual TaqMan® primer-probes for the following miRNAs: miR-10a, miR-34a, miR-494 and let-7a. Briefly, 1 µl from the diluted pre-amplified product was combined with 1 µl of TaqMan^TM^ MicroRNA Assays (20x) (Applied Biosystems, Belgium) and 10 µl of TaqMan™ PCR Master Mix (Applied Biosystems, Belgium) in a final volume of 20 µl. All reactions were run in triplicates.

### Analysis of miRNA expression levels

The data obtained by TLDA cards, Custom cards and individual QPCR Assays was analysed based on the same parameters and normalization method. MiRNAs with a Ct-value > 32 were thought to be lowly expressed and then excluded from further analysis. According to NormFinder algorithm and DataAssist™ Software v.3.01, U6 and snoR202 showed the lowest Ct variance between control and chemo-exposed samples and were chosen as reference genes for data normalization. The data analysis was conducted in pairs of samples (control-chemo exposed) for each single experiment. The expression level of each miRNA was calculated by the comparative Ct method (ΔΔCt) and the fold change (FC) was calculated by the equation 2^−ΔΔCt^. The final fold change was expressed in terms of geometric mean. The miRNAs with FC < 0.5 were considered to be down-regulated while others with FC > 1.5 were considered to be up-regulated.

### *In Silico* gene expression Analysis

We used the TargetScan (targetscan.org) and MirTarBase (mirtarbase.mbc.nctu.edu.tw) for the *in silico* prediction and identification of the genes targeted by mmu-miR-let-7a. A list of predicted and validated gene-targets was submitted to the database for annotation, visualization, and integrated discovery (DAVID) and pathway enrichment analysis was performed.

### Liposome-Mediated Transfection with synthetic miRNAs

For the *in vitro* transfection of PND3 ovaries with let-7a mimic (mirVana® miRNA mimic, Life Technologies Europe BV), we used the Lipofectamine RNAiMAX transfection reagent (Invitrogen, Life Technologies Europe BV). The transfection mix was prepared using 300pmol of let-7a mimic and 20 µl of Lipofectamine RNAiMax in 400 µl of Opti-MEM reduced-serum medium (Invitrogen, Life Technologies Europe BV) supplemented with ascorbic acid (50 μg/ml), human transferrin (27.5 μg/ml), penicillin G (5 IU/ml) and streptomycin sulphate (3.7IU/ml) (Sigma, Belgium). The transfection mix was incubated for 15 minutes at RT to form the Lipofectamine-miRNA complexes prior to transfection into ovaries. The PND3 ovaries were cultured on polycarbonate inserts using Corning® 24 Well Tissue Culture-Treated Plates (Sigma, Belgium) in 400 µl of the transfection mix for 2 days at 37 °C in a humidified incubator with 5% CO_2_.

For the assessment and optimization of the miRNA delivery, the ovaries were cultured with a fluorescent-labelled dsRNA (Alexa Fluor® 555-labeled, RNA duplex, BLOCK-iTTM Alexa Fluor® Red Fluorescent Control) (Invitrogen Life Technologies, Europe BV) and then prepared for serially cryo-sectioning (10 µm). All sections were mounted with fluoroshield mounting medium (VECTASHIELD Antifade Mounting Medium with DAPI) (Sigma, Belgium) on glass slides and observed using a Leica® DM 2000 fluorescent microscope.

After miRNA-mimic transfection, the ovaries were exposed to 4-HC (20 µM) or not for 1 hour before processing for RNA extraction or histological assessment.

### Gene expression quantification by QPCR

Specific miRNA-targeted genes expression was evaluated by QRT-PCR. The ReliaPrep™ RNA Tissue Miniprep System (Promega, Netherlands) was used for total RNA extraction according to manufacturer’s instructions. The RNA was quantified using NanoDrop spectrophotometer (Thermo Scientific, Belgium). Reverse transcription reactions were preformed using 200 ng of total RNA and random primers. For each QPCR reaction, 2 ng of cDNA were used with SYBR® Green Master mix (Applied Biosystems, Belgium) and 10 μM of gene-specific forward and reverse primers (Eurogentec, Belgium) (Supplementary Table S3). Individual samples were analysed in duplicates and the gene RPL19 was used for data normalization. The expression level of each gene was calculated by the comparative Ct method (ΔCt) and the fold change was calculated by the equation 2^−ΔCt^.

### Histology and follicular count

After 2 days of culture with or without treatments, ovaries were fixed in 4% paraformaldehyde for 2 hours at 4 °C, embedded in paraffin, and serially sectioned (5 μm). Every fifth section was stained with hematoxylin and eosin (H&E) for assessing the follicular morphology and stage based on Gougeon’s classification^2^. Only the follicles with visible oocyte nucleus were counted and characterized as primordial, transitory, primary and secondary.

### Immunohistochemistry

The Ki-67 immunostaining was used to evaluate the proliferation rates in the ovarian follicles. The procedure was performed as it was previously described^27^. The slides were incubated with the primary antibody (Ki-67 mouse α-human 1:400, BD Bioscience, Belgium 1:1000, Cell Signaling, Netherlands) overnight at 4 °C. As negative control, the primary antibody was replaced by species-adapted non-specific IgG. Sections were examined using a Leica DM 2000 fluorescent microscope.

### TUNEL assay

Apoptosis was assessed by TUNEL staining (*In Situ* Death Cell Detection Kit, Roche, Belgium). The procedure was performed as it was previously described^27^. The sections were observed using a Leica DM 2000 fluorescent microscope. The image analysis was performed using ZEN 2.3 software on at least 3 randomly selected sections per ovary from 3 individual experiments. Hoechst and TUNEL positive cells were quantified considering a lower thresholding to exclude all autofluorescence signals. To estimate the apoptotic level, TUNEL positive cells were calculated and expressed as a percentage of total Hoechst positive cells per ovary.

### Statistical analysis

All statistical analyses were performed by the IBM® SPSS Statistics 24 program. At least three biological replicates were performed for every experiment in this study and all values represented as the mean or geometric-mean ± standard error of the mean (SEM). A paired t-test was performed for normally distributed data, while non-parametric test like Mann Whitney U and Kruskal Wallis test were used to analyse the data non-normally distributed data among groups. P-value < 0.05 was defined as a statistically significant difference.

## Supplementary information

Figure S1

Supplementary Tables S1-S2-S3
