# Supplementary material for: MicroRNA profiling and identification of let-7a as a target to prevent chemotherapy-induced primordial follicles apoptosis in mouse ovaries
Source: Sci Rep. 2019 Jul 3;9:9636. doi: 10.1038/s41598-019-45642-w (PMC6610114; doi:10.1038/s41598-019-45642-w)
Supplement: Supplementary file 1 — Figure S1 [file 41598_2019_45642_MOESM1_ESM.pdf]

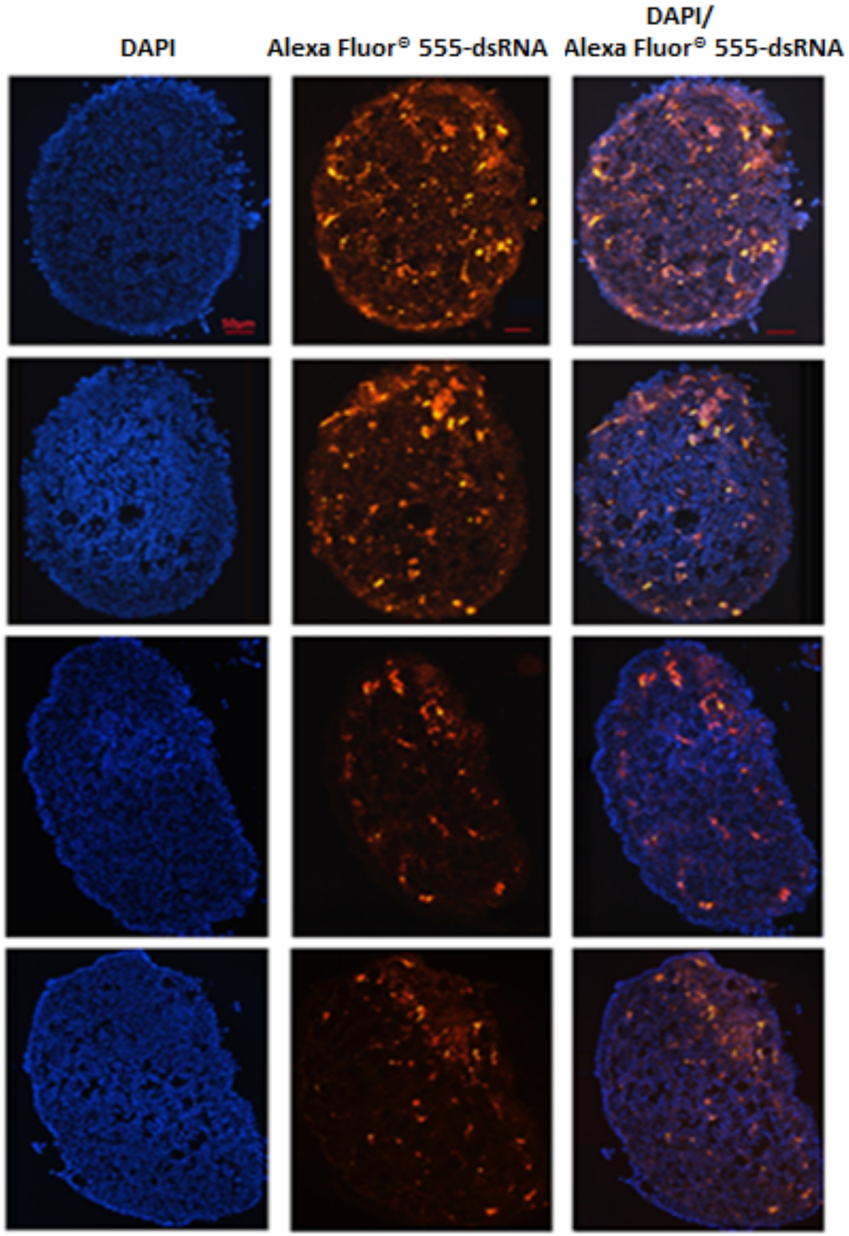

Figure S1. Representative images of PND3 ovary, after 2 days of transfection with Alexa Fluor® 555-labeled, dsRNA/ Lipofectamine RNAiMax. PND3 ovarian sections (10µm) showed nuclear labelling with Hoechst (blue) and Alexa Fluor® 555-labeled, dsRNA (red). The merged images indicate that the dsRNA distribution is not uniform but it was successfully transferred into the ovaries.
