# Supplementary material for: MicroRNA profiling and identification of let-7a as a target to prevent chemotherapy-induced primordial follicles apoptosis in mouse ovaries
Source: Sci Rep. 2019 Jul 3;9:9636. doi: 10.1038/s41598-019-45642-w (PMC6610114; doi:10.1038/s41598-019-45642-w)
Supplement: Supplementary file 2 — Supplementary Tables S1-S2-S3 [file 41598_2019_45642_MOESM2_ESM.docx]

**Supplementary data**

**Table S1.** List of the 245 genes which are expressed in PND3 ovaries and their fold change (FC) between control and chemotherapy exposed (1h/4-HC/20µM) conditions in a cohort of 6 paired samples. Fold change is expressed as the geometric mean.

| Stable Expressed Genes | Fold Change | Upregulated Genes  (FC>1.5) | Fold Change | Downregulated Genes  (FC<0.5) | Fold Change |
| --- | --- | --- | --- | --- | --- |
| mmu-let-7i | 1.17 | mmu-let-7e | 2.00 | mmu-let-7a | 0.34 |
| mmu-miR-145 | 1.18 | mmu-let-7f | 26.39 | mmu-miR-10a | 0.17 |
| mmu-miR-15b | 1.27 | mmu-let-7g | 2.19 | mmu-miR-135a | 0.41 |
| mmu-miR-181c | 1.48 | mmu-miR-101a | 6.29 | mmu-miR-15a | 0.06 |
| mmu-miR-187 | 1.40 | mmu-miR-107 | 11.90 | mmu-miR-188-5p | 0.04 |
| mmu-miR-191 | 0.97 | mmu-miR-125a-3p | 8.66 | mmu-miR-192 | 0.37 |
| mmu-miR-200a | 0.92 | mmu-miR-125a-5p | 10.22 | mmu-miR-194 | 0.14 |
| mmu-miR-200b | 0.55 | mmu-miR-125b-3p | 2.28 | mmu-miR-19b | 0.34 |
| mmu-miR-202-3p | 1.10 | mmu-miR-125b-5p | 4.61 | mmu-miR-223 | 0.10 |
| mmu-miR-20a | 0.93 | mmu-miR-127 | 3.02 | mmu-miR-25 | 0.32 |
| mmu-miR-20b | 1.02 | mmu-miR-128a | 3.85 | mmu-miR-29b | 0.06 |
| mmu-miR-210 | 1.48 | mmu-miR-130a | 2.85 | mmu-miR-29c | 0.13 |
| mmu-miR-214 | 0.97 | mmu-miR-130b | 4.77 | mmu-miR-30c | 0.44 |
| mmu-miR-215 | 0.82 | mmu-miR-132 | 2.46 | mmu-miR-324-5p | 0.33 |
| mmu-miR-221 | 1.45 | mmu-miR-134 | 1.85 | mmu-miR-329 | 0.17 |
| mmu-miR-224 | 0.70 | mmu-miR-136 | 2.78 | mmu-miR-335-3p | 0.54 |
| mmu-miR-26a | 0.65 | mmu-miR-138 | 1.57 | mmu-miR-345-3p | 0.30 |
| mmu-miR-26b | 0.62 | mmu-miR-139-3p | 7.32 | mmu-miR-375 | 0.20 |
| mmu-miR-28 | 0.85 | mmu-miR-146a | 10.82 | mmu-miR-376b | 0.43 |
| mmu-miR-292-3p | 0.76 | mmu-miR-146b | 4.21 | mmu-miR-425 | 0.03 |
| mmu-miR-293 | 1.23 | mmu-miR-148a | 3.94 | mmu-miR-434-5p | 0.25 |
| mmu-miR-296-3p | 1.00 | mmu-miR-150 | 15.15 | mmu-miR-467b | 0.21 |
| mmu-miR-298 | 0.66 | mmu-miR-152 | 3.11 | mmu-miR-495 | 0.27 |
| mmu-miR-301a | 0.53 | mmu-miR-155 | 19.91 | mmu-miR-540-3p | 0.05 |
| mmu-miR-31 | 1.16 | mmu-miR-181a | 1.74 | mmu-miR-674 | 0.36 |
| mmu-miR-340-3p | 0.59 | mmu-miR-185 | 3.37 | mmu-miR-881 | 0.22 |
| mmu-miR-345-5p | 1.06 | mmu-miR-186 | 2.57 | mmu-miR-21 | 0.38 |
| mmu-miR-34c | 0.93 | mmu-miR-195 | 1.83 | mmu-miR-322 | 0.35 |
| mmu-miR-351 | 0.97 | mmu-miR-196b | 9.14 | mmu-miR-667 | 0.21 |
| mmu-miR-370 | 0.74 | mmu-miR-197 | 3.87 | mmu-miR-883a-3p | 0.23 |
| mmu-miR-379 | 0.73 | mmu-miR-199a-3p | 4.84 | mmu-miR-451 | 0.24 |
| mmu-miR-380-5p | 0.77 | mmu-miR-19a | 5.40 | mmu-miR-376a | 0.39 |
| mmu-miR-409-3p | 1.25 | mmu-miR-222 | 3.41 | mmu-miR-376c | 0.52 |
| mmu-miR-410 | 0.97 | mmu-miR-24 | 1.74 | mmu-miR-350 | 0.41 |
| mmu-miR-429 | 1.15 | mmu-miR-294 | 3.64 | mmu-miR-344 | 0.24 |
| mmu-miR-431 | 0.86 | mmu-miR-295 | 2.66 | mmu-miR-148b | 0.28 |
| mmu-miR-465a-3p | 0.68 | mmu-miR-296-5p | 12.76 | mmu-miR-143 | 0.56 |
| mmu-miR-465a-5p | 1.22 | mmu-miR-30a | 2.43 | mmu-miR-582-5p | 0.05 |
| mmu-miR-466h | 0.52 | mmu-miR-32 | 14.81 | mmu-miR-450a-5p | 0.31 |
| mmu-miR-467a | 0.51 | mmu-miR-320 | 1.68 | mmu-miR-106b | 0.42 |
| mmu-miR-467c | 0.55 | mmu-miR-323-3p | 2.11 |  |  |
| mmu-miR-486 | 0.85 | mmu-miR-324-3p | 1.69 |  |  |
| mmu-miR-491 | 0.75 | mmu-miR-328 | 3.44 |  |  |
| mmu-miR-497 | 1.04 | mmu-miR-330 | 2.28 |  |  |
| mmu-miR-503 | 1.34 | mmu-miR-331-3p | 3.99 |  |  |
| mmu-miR-532-5p | 1.10 | mmu-miR-340-5p | 2.21 |  |  |
| mmu-miR-542-5p | 1.20 | mmu-miR-34b-3p | 4.72 |  |  |
| mmu-miR-543 | 0.88 | mmu-miR-369-5p | 1.90 |  |  |
| mmu-miR-547 | 0.51 | mmu-miR-423-5p | 2.75 |  |  |
| mmu-miR-652 | 0.90 | mmu-miR-450b-5p | 50.33 |  |  |
| mmu-miR-672 | 0.78 | mmu-miR-455 | 9.32 |  |  |
| mmu-miR-675-3p | 1.15 | mmu-miR-467d | 2.39 |  |  |
| mmu-miR-676 | 0.64 | mmu-miR-467e | 9.25 |  |  |
| mmu-miR-708 | 0.54 | mmu-miR-470 | 4.05 |  |  |
| mmu-miR-743b-3p | 1.05 | mmu-miR-484 | 1.86 |  |  |
| mmu-miR-744 | 1.02 | mmu-miR-494 | 2.46 |  |  |
| mmu-miR-872 | 0.76 | mmu-miR-501-3p | 3.19 |  |  |
| mmu-miR-883b-3p | 0.52 | mmu-miR-509-3p | 8.85 |  |  |
| mmu-miR-99a | 0.73 | mmu-miR-532-3p | 3.68 |  |  |
| mmu-miR-99b | 1.24 | mmu-miR-598 | 6.49 |  |  |
| snoRNA202 | 0.91 | mmu-miR-680 | 2.32 |  |  |
| U6 snRNA | 1.10 | mmu-miR-743a- | 5.34 |  |  |
| mmu-miR-100 | 0.80 | mmu-miR-770-3p | 3.75 |  |  |
| mmu-miR-27a | 1.16 | mmu-miR-7a | 8.00 |  |  |
| mmu-miR-291a-3p | 0.89 | mmu-miR-92a | 2.31 |  |  |
| mmu-miR-335-5p | 0.74 | U87 | 4.62 |  |  |
| mmu-miR-34a | 0.95 | mmu-miR-16 | 8.00 |  |  |
| mmu-miR-383 | 0.72 | mmu-miR-204 | 1.53 |  |  |
| mmu-miR-433 | 0.95 | mmu-miR-18a | 1.68 |  |  |
| mmu-miR-434-3p | 0.90 | mmu-miR-193b | 2.69 |  |  |
| mmu-miR-500 | 0.86 | mmu-miR-301b | 2.01 |  |  |
| mmu-miR-682 | 0.81 | mmu-miR-30d | 2.09 |  |  |
| mmu-miR-666-5p | 0.64 | mmu-miR-30e | 2.02 |  |  |
| Y1-001727 | 0.47 | mmu-miR-465b-5p | 1.57 |  |  |
|  |  | mmu-miR-487b | 1.36 |  |  |
|  |  | mmu-miR-546 | 3.89 |  |  |
|  |  | mmu-miR-574-3p | 1.94 |  |  |
|  |  | mmu-miR-93 | 7.27 |  |  |
|  |  | mmu-miR-103 | 10.80 |  |  |
|  |  | mmu-miR-23b | 3.80 |  |  |
|  |  | mmu-miR-331-5p | 12.14 |  |  |

**Table S2.** List of the 21 selected miRNAs on Custom TLDA cards. The table presents the fold change of miRNAs expression after 1 and 24h exposure to 4-HC/20µM. The fold change is presented as the geometric mean. The data was analysed using paired t-test, p-value< 0.05.

| **Target Name** | **Fold Change**  **1h/4-HC/20µM** | **P-value** | **Fold Change**  **24h/4-HC/20µM** | **P-value** |
| --- | --- | --- | --- | --- |
| miR-16 | 0.25 | 0.0168 | 0.90 | 0.768 |
| miR-27a | 0.53 | 0.1290 | 0.79 | 0.178 |
| miR-34a | 0.28 | 0.0057 | 0.41 | 0.037 |
| miR-107 | 0.35 | 0.0620 | 0.93 | 0.653 |
| miR-125a-5p | 0.48 | 0.0654 | 1.72 | 0.323 |
| miR-145 | 0.51 | 0.0259 | 0.82 | 0.089 |
| miR-146a | 1.12 | 0.8063 | 1.18 | 0.581 |
| miR-15a | 0.29 | 0.3857 | 0.33 | 0.063 |
| miR-150 | 1.36 | 0.1315 | 0.61 | 0.152 |
| miR-181c | 0.79 | 0.3170 | 1.13 | 0.831 |
| miR-200b | 0.41 | 0.1070 | 0.90 | 0.606 |
| miR-223 | 0.20 | 0.0457 | 0.50 | 0.170 |
| miR-320 | 0.97 | 0.9730 | 0.77 | 0.248 |
| miR-383 | 0.95 | 0.9676 | 0.80 | 0.633 |
| miR-10a | 0.34 | 0.0060 | 0.37 | 0.000 |
| miR-425-5p | 1.23 | 0.2627 | 0.31 | 0.027 |
| miR-484 | 0.83 | 0.4999 | 0.80 | 0.037 |
| miR-494 | 0.48 | 0.0248 | 0.65 | 0.020 |
| miR-544 | 0.91 | 0.5498 | 0.83 | 0.789 |
| let-7a | 0.15 | 0.0111 | 1.06 | 0.989 |
| miR-29a | 0.48 | 0.2519 | 1.27 | 0.927 |

**Table S3.** List of the primers and their sequences used for gene expression analysis.

| **Primers** | **Sequence** |
| --- | --- |
| FasL_Forward | CTG-GGT-TGT-ACT-TCG-TGT-ATT-CC |
| FasL_Reverse | TGT-CCA-GTA-GTG-CAG-TAG-TTC-AA |
| STAT3_Forward | TGA-AGG-TGG-TGG-AGA-ACC-TC |
| STAT3_Reverse | GCT-GCT-GCA-TCT-TCT-GTC-TG |
| HMGA2_Forward | CAA-GAG-GCA-GAC-CTA-GGA-AAT-G |
| HMGA2_Reverse | GAT-CCA-ACT-GAT-GCT-GAG-GTA-G |
| CASP3_Forward | ATC-ATT-CAG-GCC-TGC-CGG-GGT |
| CASP3_Reverse | GGA-CTG-GAT-GAA-CCA-CGA-CCC-G |
| BAX_Forward | CAA-GAC-CAG-GGT-GGC-TGG-GAA-G |
| BAX_Reverse | AGA-CAC-AGT-CCA-AGG-CAG-TGG-GA |
| BCL2_Forward | TTG-TAA-TTC-ATC-TGC-CGC-CG |
| BCL2_Reverse | AGG-GTT-TCC-AGA-TTG-GGT-CC |
| TLR5_Forward | CAC-ACC-CTT-AGC-ATG-AGC-CT |
| TLR5_Reverse | GGG-GAA-CTC-AGC-ATC-CAT-CC |
| RPL19_Forward | GAA-AGG-TGC-TTC-CGA-TTC-CA |
| RPL19_Reverse | TGA-TCG-CTT-GAT-GCA-AAT-CC |
| RPTOR_Forward | AAT-GCT-GGC-CTC-ATC-GTC-AA |
| RPTOR_Reverse | CAT-CTG-GGC-AAG-TGG-ATG-GT |
| mTOR_Forward | ATC-CAG-ACC-CGT-AAC-CTC-CA |
| mTOR_Reverse | ACA-GGC-ACC-CAT-CCA-ATC-TG |
| KITL_Forward  KITL_Reverse | TGGTGGCAAATCTTCCAAAT  TTCTTCGGTGCGTTTTCTTC |
